# Early Detection of Alzheimer's Disease and Related Dementias From Spontaneous Speech Using Foundation Speech and Language Models: Comparative Evaluation

**DOI:** 10.2196/79411

**Published:** 2026-05-13

**Authors:** Jingyu Li, Lingchao Mao, Xi Mao, Hairong Wang, Zhendong Wang, Xuelei Sherry Ni

**Affiliations:** 1 H. Milton Stewart School of Industrial and Systems Engineering Georgia Institute of Technology Atlanta, GA United States; 2 Department of Economics The University of Texas Rio Grande Valley Edinburg, TX United States; 3 Operations Research and Industrial Engineering, Cockrell School of Engineering The University of Texas at Austin Austin, TX United States; 4 Shandong Provincial Key Medical and Health Laboratory of Digital Psychiatry Shandong Mental Health Center Jinan China; 5 School of Data Science and Analytics Kennesaw State University Marietta, GA United States

**Keywords:** Alzheimer's disease, clinical screening, comparative evaluation, dementia detection, foundation speech models, language models, speech biomarkers, spontaneous speech analysis

## Abstract

**Background:**

Alzheimer's disease and related dementias (ADRD) are progressive neurodegenerative conditions where early detection is critical for timely intervention and care planning. However, current diagnostic methods are often inaccessible, costly, and delayed, especially for underserved populations. There is a growing need for scalable, noninvasive tools that can support timely diagnosis. Spontaneous speech contains rich acoustic and linguistic markers that can serve as noninvasive behavioral markers for cognitive decline. Foundation models, pretrained on large-scale audio or text data, generate high-dimensional embeddings that encode rich contextual and acoustic information.

**Objective:**

This study benchmarks open-source foundation language and speech models to evaluate their effectiveness in detecting ADRD from spontaneous speech as a potential solution for early, noninvasive, and scalable ADRD detection.

**Methods:**

In this study, we used the Pioneering Research for Early Prediction of Alzheimer’s and Related Dementias EUREKA (PREPARE) Challenge dataset, which consists of audio recordings from over 1600 participants with 3 distinct categories of cognitive decline: healthy control (HC), mild cognitive impairment (MCI), and Alzheimer's disease (AD). We further excluded samples that are non-English, nonspontaneous speech, or of poor quality. Our final samples included 703 (59.13%) HC, 81 (6.81%) MCI, and 405 (34.06%) AD cases. We systematically benchmarked 18 open-source foundation speech and language models to classify cognitive status into 3 categories (HC, MCI, or AD). Post hoc interpretability analysis was performed for the best-performing model using Shapley additive explanations linking high-dimensional embeddings with explainable acoustic and linguistic markers.

**Results:**

Whisper-medium model achieved the highest performance among speech models at 0.731 accuracy and 0.802 area under the curve, while Bidirectional Encoder Representations from Transformers with pause annotation achieved the top accuracy of 0.662 and 0.744 area under the curve among language models. Overall, ADRD detection based on state-of-the-art automatic speech recognition model-generated audio-embeddings outperformed other models, and the inclusion of nonsemantic information, such as pause patterns, consistently improved the classification performance of text-embedding–based models.

**Conclusions:**

Our work presents a comprehensive comparative evaluation of state-of-the-art speech and language models for AD and MCI detection on a large, clinically relevant dataset. Embeddings derived from acoustic models, which capture both semantic and acoustic information, show promising performance and highlight the potential for developing a more scalable, noninvasive, and cost-effective early detection tool for ADRD.

## Introduction

### Alzheimer Disease and Early Detection

Alzheimer disease and related dementias (ADRD) are progressive brain disorders that gradually impair memory, thinking, and daily functioning. ADRD typically progresses through 3 stages: healthy control (HC) or preclinical, mild cognitive impairment (MCI), and formal Alzheimer disease (AD) or dementia [1]. MCI, while not always a precursor to AD, is an important early indicator and target for intervention [2].

Because ADRD progresses slowly and irreversibly, and new US Food and Drug Administration–approved drugs like lecanemab and donanemab are effective only in early stages, timely detection is essential to delay symptoms and expand treatment options [3,4]. Although advances in brain imaging, blood biomarkers, and artificial intelligence have improved early detection tools, global health care systems remain underequipped. For instance, limited access to positron emission tomography/magnetic resonance imaging and dementia specialists leads to projected diagnostic delays of up to 10 years in the United Kingdom [5]. A 6-country study found that less than half of individuals who reported memory problems received a formal diagnosis, and few underwent advanced testing—even when seeing a specialist [6]. In the United States, early detection for ADRD is also inadequate and often delayed. Only 8% of Medicare enrollees receive a diagnosis of MCI, and more than 99% of primary care providers and clinics are identifying fewer cases than expected. Even specialists such as geriatricians missed over 80% of early cases [7]. Estimates suggest that 14% of MCI cases progress to dementia annually [8], and over 7 million Medicare beneficiaries may miss treatment opportunities [9]. Missed and late diagnoses often disproportionately affect underrepresented populations, such as racial and ethnic minorities, rural communities, and those with lower socioeconomic status. The worsening health disparities in Alzheimer care and outcomes highlight the critical need for accessible and scalable diagnostic solutions to support early detection of ADRD [10,11].

### Language Analysis for ADRD Detection

To close the gap between need and access, health care systems are exploring tools that support earlier, faster, and more affordable ADRD detection. One promising direction involves analyzing paralinguistic and linguistic patterns of speech, since language is among the earliest and most visible domains affected by ADRD [12,13].

Common language symptoms in patients with AD include naming difficulties, repetition, vague word use, inappropriate pronouns, etc [14,15]. These often lead to fluent yet uninformative speech, with reduced coherence and incomplete sentences [16]. Temporal speech changes such as increased hesitations and slower tempo can also occur, reflecting cognitive challenges in planning and organizing language [17,18].

Accordingly, linguistic and paralinguistic features have been widely studied as noninvasive behavioral markers of ADRD [19-21]. Markers such as semantic fluency, speech recognition, and acoustic features have proven effective in distinguishing normal aging from early MCI [21-23]. Integrating these tools into telehealth workflows, for example, through virtual memory testing and teleneuropsychology, offers a promising approach for remote screening and patient triaging. Such services have been well-received by patients and have helped reduce diagnostic delays [24,25].

### Modern Foundation Speech and Language Models for ADRD Detection

Traditional feature engineering approaches for ADRD detection have relied on manually engineered features (eg, lexical diversity, syntactic complexity, and coherence from transcripts) and acoustic features (eg, pause duration, reaction time, loudness, and speech rate) [19-21,23,26,27]. These features are then fed into machine learning classifiers. However, these pipelines require manual feature engineering, and their performance can depend on controlled environments where speech samples are collected. Robin et al [22] emphasized the importance of rigorously evaluating recording quality and data processing accuracy. Recent advances in deep learning, especially in natural language processing, have led to highly capable foundation speech and language models. These models are pretrained on large-scale datasets. These models learn to capture rich contextual information and produce high-dimensional representations of raw speech or texts, known as “embeddings,” thus simplifying the labor-intensive and human-dependent preprocessing step. These embeddings capture semantic, syntactic, and prosodic signals and have shown promise in ADRD detection [28-30]. Embeddings generated by large language and speech models also demonstrate greater flexibility, robustness to varied inputs, and even multilingual capabilities. This shift supports more generalizable and scalable detection in telehealth or low-resource settings. Shakeri and Farmanbar [13] provide a detailed review of the natural language processing in AD research in their 2025 paper.

Two common approaches exist for extracting these embeddings for ADRD detection from deep learning models. One approach is to transcribe speech to text and then use a language model to extract text embeddings [28,29]. However, the transcript-based approach captures only semantic information, while neglecting important nonsemantic information such as paralinguistic features and pause patterns [29,30]. To address these gaps, some researchers have manually annotated speech pauses in transcripts to preserve nonsemantic information contained in patients’ speech data [15,29,30]. This step has been shown to improve prediction accuracy, but it introduces additional complexity, lacks flexibility, and captures limited features of speech. Therefore, current research has been limited to binary ADRD detection using small or curated datasets.

An increasingly promising alternative is to extract embeddings directly from raw audio using pretrained automatic speech recognition (ASR) models trained for both recognition and representation tasks. These models preserve both semantic and paralinguistic cues without requiring transcription. Among them, OpenAI’s Whisper has demonstrated state-of-the-art performance across varied languages and acoustic conditions [16]. However, despite their potential, these models have not yet been rigorously benchmarked for ADRD detection in large and real-world speech datasets.

### Objective

In this study, we conduct a comprehensive comparative evaluation of modern foundation speech and language models for ADRD detection. Rather than developing advanced models to achieve higher prediction accuracy, the goal of this study is to establish a comprehensive benchmark by systematically evaluating a range of open-source foundation models and comparing their baseline performance in ADRD detection on a large clinical dataset. These results offer an initial reference point for applying these toolkits to ARDR detection.

Specifically, we compare ADRD detection based on text and audio embeddings generated by 18 state-of-the-art open-source language and speech models. We evaluate whether the high-dimensional embeddings generated by these models can serve as effective behavioral markers for ADRD detection. To the best of our knowledge, neither the Bidirectional Encoder Representations from Transformers (BERT) family models nor the speech models evaluated in this study have been systematically and comprehensively assessed for ADRD detection on a large-scale dataset. Our experiments use the Pioneering Research for Early Prediction of Alzheimer’s and Related Dementias EUREKA (PREPARE) Challenge dataset—a large, diverse, and publicly available speech corpus curated from DementiaBank containing over 1600 samples to ensure relevance to a diverse population. Notably, our findings identify the medium-sized Whisper model as the top performer in ADRD detection from speech and demonstrate its advanced language understanding to capture linguistic and acoustic markers associated with ADRD. We further discuss an acoustic-based ADRD detection pipeline with strong potential to advance early detection and intervention compared to the traditional diagnosis timeline (Figure S1.1 in Multimedia Appendix 1 [31-34]). This study highlights the promise of foundation speech models for early ADRD detection and offers practical guidance for the development of scalable and noninvasive ADRD screening tools.

## Methods

### The PREPARE Challenge Audio Data

The audio data used in this study are available as part of the PREPARE Phase 2 Challenge-Acoustic Track [35]. This dataset was curated from the DementiaBank [36], a well-established clinical research repository containing multilingual audio recordings of participants performing a wide range of spontaneous speech tasks, including picture description, verbal fluency, and sentence construction. Participants contributing to DementiaBank are recruited through affiliated memory clinics and research sites and, in some cases, community outreach/promotional materials. All contributed datasets involve institutional review board’s (IRB) approval and informed consent, as specified in the TalkBank/DementiaBank website [37]. Diagnostic labels (HC, MCI, or AD) are assigned using established clinical criteria. The PREPARE consortium subsequently curated these data, ensuring deidentification and creating consistent metadata before public release.

The original curated dataset consists of audio recordings from 2086 participants, divided into 1646 training samples and 412 testing samples during the PREPARE Phase 2 Challenge. In this study, we used only the provided training dataset, as the cognitive status labels for the testing data were not released.

The dataset comprised speech samples from multiple languages and various language tasks, including sentence reading and spontaneous speech tasks. In the spontaneous speech task, participants were asked to describe the Cookies Theft picture for English speakers, which is a popular language impairment assessment tool originally used in the Boston Diagnostic Aphasia Examination protocol [17]. Since speech and language models primarily analyze speech content, we focused our analysis exclusively on spontaneous speech samples. To identify spontaneous speech samples, we applied density-based spatial clustering of applications with noise on BERT embeddings of the speech transcriptions, followed by human evaluation to classify clusters to language tasks [18,38]. See more details in Figure S2.1 in Multimedia Appendix 1. The transcription and language of recordings were detected using the Whisper-small model [16].

**Figure 1:**
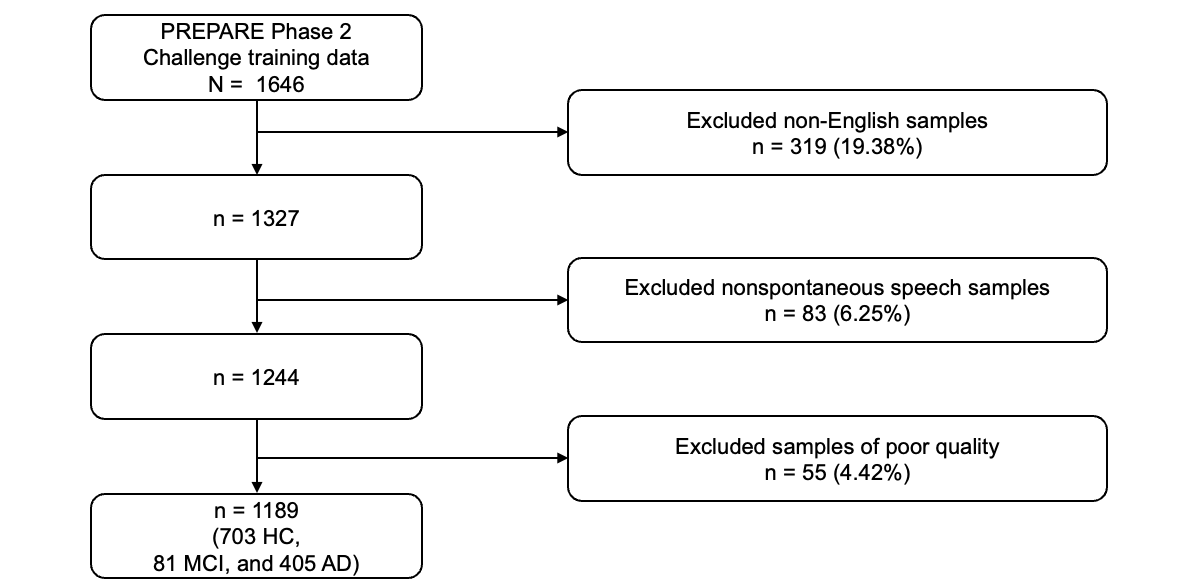
Data inclusion criteria. Speech samples were excluded if they were non-English, nonspontaneous, or of poor audio quality. The final dataset included 1189 samples: 703 from healthy controls (HC), 405 from individuals with Alzheimer disease (AD), and 81 from individuals with mild cognitive impairment (MCI).

Given that the majority of the recordings were in English, we excluded 319 non-English samples. Additionally, we removed 55 recordings of poor quality, defined as those with participant speech lasting less than 3 seconds or excessive background noise, based on human review. Demographic information of each participant, including age and gender, is also available. A detailed, step-by-step description of the data inclusion process is shown in Figure 1. The final dataset comprised recordings from 1189 participants (≤30 seconds each), including 703 classified as HC, 81 as MCI, and 405 as AD. The final sample (n=1189) included 680 female and 509 male individuals, with a mean age of 75.178 (SD 8.430) years.

### Modeling Pipelines

#### Overview

**Figure 2:**
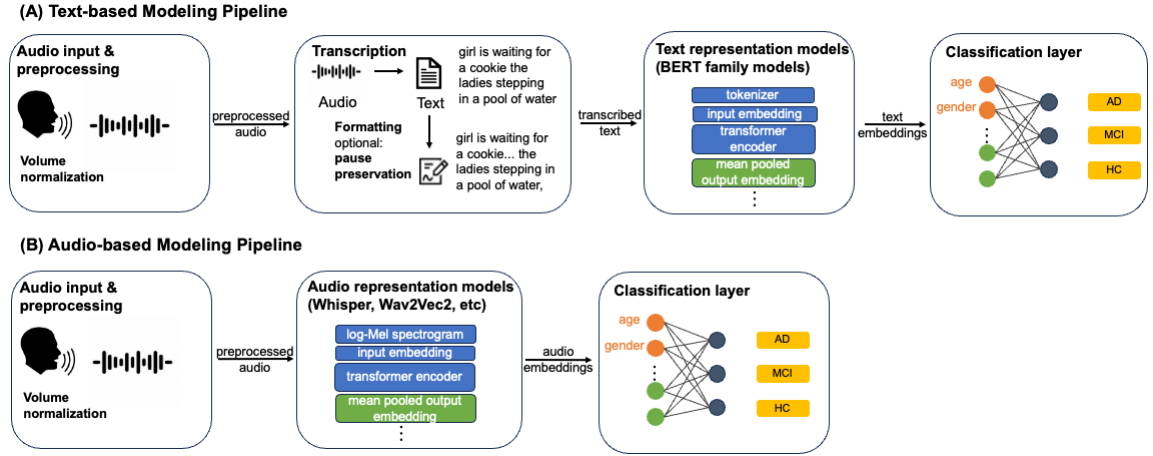
Overview of the modeling pipeline for Alzheimer disease and related dementias detection from spontaneous speech samples. (A) Text-based Modeling Pipeline: the system takes and preprocesses audio input, transcribes audio into text (optional: pause is annotated), extracts sentence-level embeddings from transcribed text using a text representation model, and feeds embeddings into a neural network to determine patient’s cognitive status into 3 categories: healthy control (HC), mild cognitive impairment (MCI), or Alzheimer disease (AD). (B) Audio-based Modeling Pipeline: the system takes and preprocesses audio input, extracts sentence-level embeddings from audio data directly using an audio representation model, and feeds embeddings into a neural network to determine the patient’s cognitive status. The extracted embeddings are concatenated with demographic information before feeding them into the classification layer. BERT: Bidirectional Encoder Representations from Transformers.

We explore two deep learning–based pipelines to extract embeddings from foundation models and classify cognitive status of the participants from audio recordings: (1) a *text-based pipeline*, where a language model analyzes the transcribed content of the speech to identify linguistic patterns associated with cognitive decline and (2) an *audio-based pipeline*, where a speech model transforms audio signals into embeddings that capture both the linguistic and acoustic patterns characteristic of ADRD. Figure 2 shows the detailed workflow for both pipelines.

To ensure the comprehensiveness of our benchmarking study, we compared the performance of the 2 deep learning–based pipelines with that of the traditional feature engineering pipeline, where classification models were trained on widely used preextracted paralinguistic and linguistic features reported in the literature including Geneva Minimalistic Acoustic Parameter Set (eGeMAPS) [31], Computational Paralinguistics Challenge [32], Mel-Frequency Cepstral Coefficients [39], and the Linguistic Inquiry and Word Count [33]. Detailed description of these 4 groups of features can be found in Table 1 and Multimedia Appendix 2. More details about this pipeline are provided in section S3 of the Multimedia Appendix 1.

**Table 1:** Traditional acoustic and linguistic features.

Feature set	Features, n	Acoustic and linguistic variables
eGeMAPS^a^ [31]	89	eGeMAPS features provide a compact set of acoustically and physiologically motivated speech descriptors, including pitch, loudness/intensity, spectral shape, formant frequencies, voice quality measures (jitter, shimmer, and HNR^b^), harmonic amplitude ratios, formants, and temporal dynamics.
ComParE^c^ [32]	66	ComParE features provide a comprehensive representation of speech by capturing pitch and phonation, voice perturbations (jitter and shimmer), periodicity and noisiness (harmonics-to-noise ratio and harmonicity), loudness and amplitude, temporal dynamics (zero-crossing rate), and spectral energy distribution.
LIWC^d^ [33]	108	LIWC features capture a wide range of linguistic and psychological dimensions from text. It includes scores quantifying analytic thinking, social status or confidence, authenticity, emotional tone, as well as usage of terms related to psychological processes, emotion, cultural and personal dimensions, health, and personal needs or drives.
MFCC^e^ [34]	20	MFCCs capture the perceptual spectral envelope of speech by summarizing the distribution of energy across frequency on the Mel scale. Lower-order MFCCs reflect coarse spectral structure (eg, spectral tilt and formant distribution), while higher-order coefficients capture fine spectral details and rapid variations.

^a^eGeMAPS: Geneva Minimalistic Acoustic Parameter Set.

^b^HNR: harmonics-to-noise ratio.

^c^ComParE: Computational Paralinguistics Challenge.

^d^LIWC: Linguistic Inquiry and Word Count.

^e^MFCC: Mel-Frequency Cepstral Coefficient.

Our objective is to systematically evaluate a broad set of emerging open-source speech and language models and establish baseline performance benchmarks of these models in ADRD detection. Our central research question is whether deep learning–based pipelines, particularly the audio-based pipeline with state-of-the-art models, outperform the traditional feature engineering pipeline that has been commonly used in previous literature. In this context, we define a “strong” result as one in which a model demonstrates superior performance relative to other benchmarked models and achieves comparable or higher levels of discrimination that are considered clinically meaningful in the literature. Our benchmarks can serve as an initial reference point for future methodological improvements and a foundation for developing practical tools to be used in downstream clinical applications [13,28,29,40-42].

#### Text-Based Pipeline

##### Overview

For the text-based pipeline, we first transcribed the preprocessed audio recordings into text using an ASR system, followed by the extraction of embeddings using pretrained language models for downstream classification. This approach emphasizes the linguistic content of speech for dementia diagnosis and can benefit from the abundance of textual data available on the internet, public datasets, and open-domain corpora. To incorporate acoustic characteristics into text embeddings, we further encoded pauses manually within the transcribed text to generate pause-aware embeddings.

##### Audio Preprocessing

Each raw audio clip (48 kHz) was resampled to 16 kHz, as higher frequencies in the audio recordings typically do not contain relevant speech. Audio amplitudes were normalized, and the beginning and end silence were trimmed. Because recordings were collected in various acoustic environments, we used DeepFilterNet to suppress background noise and enhance audio clarity [43].

##### Audio Transcription

We used OpenAI’s Whisper model to transcribe audio recordings into text [16]. Preprocessed audio files were loaded and transformed into log-Mel spectrograms, which were fed into the Whisper model for transcription. Encoding pauses in the transcripts has shown superior predictive power in identifying AD status from audio data [29]. To capture speech disfluencies relevant to cognitive decline, we included an optional step of annotating word pause and sentence pause in the transcripts. Specifically, word pauses were detected by measuring the time gap between consecutive words within a segment, and a pause marker was inserted if the duration exceeded a predefined threshold of 0.05 seconds. Sentence pauses were identified by calculating the silence duration between adjacent transcription segments, with pauses above the threshold labeled accordingly. After transcription, the text underwent further preprocessing, including formatting and tokenization, to ensure compatibility with text representation models. Specifically, we removed punctuation, adjusted text casing based on model requirements, and recoded word and sentence pauses for transcripts with the optional pause annotation. Similar to the approach introduced in Yuan et al [29], we categorized pauses into 3 bins: short (under 0.5 seconds); medium (0.5-2 seconds); and long (over 2 seconds), which were represented using punctuation marks—commas (,), periods (.), and ellipses (...), respectively to represent different levels of disfluencies in speech. The processed text underwent proper tokenization before being served as input to text representation models.

##### Text Embedding Models

BERT [38] and BERT-like models have been highly effective for a wide range of natural language processing tasks, including AD classification. BERT is a transformer-based language model designed to learn contextualized word representations by leveraging bidirectional self-attention to capture associations among words. Each attention head processes elements within a sequence and generates a new sequence by computing a weighted sum of the transformed input representations. The base model consists of 12 layers, 12 attention heads, and 110 M parameters, while the larger version has 24 layers, 16 attention heads, and 340 M parameters. We also included 2 BERT models adapted for the medical domain in our benchmarking study. BioBERT [44] is a domain-specific adaptation of BERT, pretrained on large-scale biomedical corpora, including PubMed abstracts and full-text articles. We also evaluated BioClinicalBERT, a model further pretrained on clinical notes [45]. While BioBERT and BioClinicalBERT retain the transformer architecture and bidirectional pretraining approach of the base version of BERT, their exposure to domain-specific corpora allows the model to generate more relevant embeddings for medical and clinical applications. In our implementation, mean-pooled sentence-level embeddings were used for downstream classification.

#### Audio-Based Pipeline

##### Overview

For the audio-based pipeline, we directly extracted speech embeddings from the recordings using foundation speech models designed from speech processing. This approach benefits from capturing phonetic, prosodic, and acoustic features relevant to speech impairments associated with dementia diagnosis.

##### Audio Preprocessing

Each raw audio clip (48 kHz) was resampled to 16 kHz, matching the training input of most audio models, and audio amplitudes were normalized. Clips were then padded or truncated to an equal length of 30-second segments. The preprocessed audio inputs were passed into model encoders to obtain mean-pooled embeddings for each clip. For Whisper models, we converted each resampled clip into 80-channel (Whisper-medium or smaller) or 128-channel (Whisper-large or larger) log-Mel spectrogram representation on 25-millisecond windows with a stride of 10 milliseconds, following the original preprocessing of OpenAI [16].

##### Audio Embedding Models

We used a range of open-source ASR models and self-supervised speech models to learn meaningful speech representations from audio data. Whisper [16] is a state-of-the-art open-source transformer-based ASR model, trained on a large-scale multilingual dataset. It consists of an encoder-decoder architecture where the encoder processes raw audio into latent representations of audio features, and the decoder generates transcriptions. The family of Whisper models varies in size, with the larger versions offering improved transcription accuracy at the cost of higher computational demand. In our study, we demonstrated cases of using both encoder and decoder outputs, where the encoder output served as embedded features, and the decoder output served as audio transcripts for further processing.

We also compared a few prior open-source deep learning models for speech representation. Wav2Vec2 [46] learns speech representations through a 2-stage training process: a multilayer convolutional neural network feature extractor encodes raw speech into latent representations, followed by a transformer encoder that refines these representations via masked speech prediction. HuBERT [47] uses a masked prediction approach where it learns to predict cluster-based speech units from unlabeled audio. This self-supervised learning method allows the model to capture speech structures without requiring transcriptions. Unispeech [48] improves upon prior models such as Wav2Vec2 and HuBERT by integrating both unlabeled speech pretraining and supervised fine-tuning within a single model. WavLM [49] extends the Wav2Vec2 framework by incorporating denoising and masked speech modeling objectives in pretraining. Data2Vec further generalizes self-supervised learning by predicting latent representations instead of specific speech tokens, thus creating a more unified architecture across multiple modalities, including speech [50].

#### Classifier and Covariates

Both pipelines were evaluated and compared on their ability to accurately classify AD. We mean-pooled sentence-level embeddings to form clip-level embeddings. We incorporated the patient’s demographic information, including age and sex, by concatenating these 2 scalars to the final encoder embeddings of all the models. These modified embeddings then served as input features to a single-layer feedforward neural network, which was trained to predict patients’ cognitive status: HC, MCI, or AD.

### Experiment Setup

#### Model Architecture and Hyperparameters

For the text-based pipeline, we used Whisper-small and Whisper-medium for transcription. We did not consider larger Whisper models, as transcripts generated by Whisper-medium did not yield performance improvements over those by Whisper-small. We used pretrained BERT (uncase-base), BioBERT, and BioClinicalBERT to extract embeddings from transcribed text. Each transcript was tokenized using BERT’s default tokenizer. The text was padded to a max length of 512 tokens or truncated if too long. The final embedded features extracted from these 3 BERT family of models are of shape (768).

For the audio-based pipeline, we evaluated 15 deep learning models designed for speech processing: Whisper (tiny, base, small, medium, and large) [16], WavLM (base, base-plus, and large) [49], Wav2Vec2 (base and large) [46], HuBERT (base and large) [47], Unispeech (large) [48], and Data2VecAudio (base and large) [50]. The extracted embeddings varied in dimensionality, with Whisper-tiny embeddings of shape (512); Whisper-base, small, WavLM, Wav2Vec2, and HuBERT embeddings of shape (768); Whisper-medium, Unispeech, and Data2VecAudio embeddings of shape (1024); and Whisper-large embeddings of shape (1280). The models were pretrained on different data corpora, from the 960-hour LibriSpeech dataset [51] used by (Wav2Vec2, HuBERT, and Data2Vec), to more diverse multisource pretraining corpora used by WavLM, and larger datasets such as the 680,000-hour combination of public and proprietary speech data used by Whisper.

For both pipelines, we used a classification layer consisting of a single-layer feedforward neural network of hidden layer size 80 for tiny to medium speech models and of hidden layer size 128 otherwise.

Cross-entropy loss was used for the multiclass classification. During training, the embedding extractors were kept frozen, and only the classification layer was trained. Models were trained for up to 100 epochs with a batch size of 32, using the Adam optimizer with a learning rate of 0.0005 and ReduceLROnPlateau scheduler. To prevent overfitting, we terminated the training if the validation loss failed to improve for 5 consecutive epochs. All pretrained models were sourced from the HuggingFace Transformers library and trained using PyTorch on an NVIDIA V100 GPU. The data preprocessing steps were performed using the pandas, numpy, audiofile, and librosa packages in Python. Evaluation metrics were computed using the sklearn and torchmetrics packages in Python.

#### Evaluation

The dataset was randomly divided into 80% training and 20% testing based on stratified sampling on the patients’ labels of cognitive status to ensure balanced class proportions. Due to class imbalance, we also oversampled minority classes (AD and MCI in our case) in the training set to ensure balanced class distribution before training. A total of 20% (2/10) of the training set was used for validation. The train-test split was repeated 5 times with different random seeds. We selected metrics commonly used in classification tasks. Accuracy provides an intuitive measure of overall classification performance and is well-suited in multiclass settings. Area under the curve (AUC), unlike other metrics, offers a threshold-independent evaluation of the model’s performance, which is important for imbalanced clinical datasets. In this 3-class classification setting, AUC was computed using a one-versus-rest macroaverage approach for each class. For each metric, we reported the mean and SD on the test set across 5 repetitions.

### Interpretability Analysis

As deep learning models often function as black boxes, we conducted a post hoc analysis to enhance the interpretability of our results. We first applied Shapley additive explanations (SHAP) [52] to identify embedding dimensions that were most contributing to predicting each of HC, MCI, and AD classes. To interpret the high-dimensional embeddings, we curated a complementary feature list comprising explainable acoustic and linguistic features (eGeMAPS and Linguistic Inquiry and Word Count), widely adopted in prior studies (Table 1). We then examined the extent to which each top contributing embedding dimension can be explained by these features by mapping these interpretable acoustic features to each embedding. Specifically, we fitted multivariate linear regression models with embeddings as the dependent variable and the curated feature set as independent variables. For each class, we identified features with a statistically significant relationship with each top contributing embedding dimension to interpret its acoustic and linguistic properties. The full list of acoustic and linguistic features and their definition can be found in Multimedia Appendix 2.

### Ethical Considerations

This study used open-access, deidentified participant recordings curated from the DementiaBank repository. Original data collection was conducted independently by the contributing institutions under institutional ethical approval and informed consent [37]. Because this research involved only secondary analysis of publicly available, deidentified data, it was determined not to involve human subjects research under the IRB policies of collaborating institutions and in accordance with US federal regulations (45 CFR 46.102). Accordingly, IRB review and approval were not required. All analyses were performed using deidentified data, and no attempt was made to reidentify participants.

## Results

### Text-Based Pipeline

Table 2 shows the results of BERT, BioBERT, and BioClinicalBERT using transcriptions generated by Whisper-small. We reported average prediction accuracy and AUC across 5 repetitions. Among all configurations, BERT-base with pause annotations on Whisper-small transcriptions achieved the highest accuracy of 0.662 (SD 0.013), while the same model using Whisper-medium transcription achieved the highest AUC of 0.744 (SD 0.014). This suggests that for the same type of model and configuration, incorporating pause annotations consistently led to improved performance in both accuracy and AUC compared to their counterparts without pause annotations. Results of the same BERT family models using transcriptions generated by Whisper-medium are reported in Multimedia Appendix 1 (S.6.1). We did not observe consistent performance gains from using a larger transcription model (Whisper-medium vs Whisper-small).

**Table 2:** Test results for text- and audio-based models across 5 replications.

Models			Data size^a^		Model size		Accuracy, mean (SD)		AUC^b^, mean (SD)
**Text-based pipeline^c^**									
	BERT^d^ without pause	3.3 B		110 M		0.657 (0.043)		0.719 (0.026)	
	BERT with pause	3.3 B		110 M		0.662 (0.013)		0.726 (0.016)	
	BioBERT without pause	18 B		110 M		0.612 (0.033)		0.720 (0.013)	
	BioBERT with pause	18 B		110 M		0.648 (0.007)		0.729 (0.011)	
	BioClinicalBERT without pause	0.5 B		110 M		0.626 (0.039)		0.713 (0.023)	
	BioClinicalBERT with pause	0.5 B		110 M		0.650 (0.021)		0.712 (0.022)	
**Audio-based pipeline**									
	Whisper-tiny.en	680,000 h		39 M		0.673 (0.039)		0.759 (0.010)	
	Whisper-base.en	680,000 h		74 M		0.707 (0.026)		0.801 (0.020)	
	Whisper-small.en	680,000 h		244 M		0.701 (0.033)		0.791 (0.018)	
	Whisper-medium.en	680,000 h		769 M		0.731 (0.020)		0.802 (0.014)	
	Whisper-large	680,000 h		1.55 B		0.702 (0.028)		0.779 (0.014)	
	Unispeech-large-1500h-cv	1500 h		94 M		0.659 (0.023)		0.737 (0.014)	
	WavLM-base	960 h		94 M		0.653 (0.013)		0.755 (0.032)	
	WavLM-base-plus	94,000 h		94 M		0.663 (0.038)		0.748 (0.018)	
	WavLM-large	94,000 h		316 M		0.694 (0.016)		0.776 (0.023)	
	Wav2Vec2-base	960 h		95 M		0.622 (0.028)		0.720 (0.018)	
	Wav2Vec2-large	960 h		137 M		0.642 (0.021)		0.694 (0.030)	
	HuBERT-base	960 h		95 M		0.683 (0.027)		0.752 (0.031)	
	HuBERT-large	960 h		317 M		0.645 (0.025)		0.725 (0.011)	
	Data2Vec-audio-base	960 h		94 M		0.559 (0.062)		0.532 (0.025)	
	Data2Vec-audio-large	960 h		317 M		0.629 (0.032)		0.667 (0.030)	

^a^Training data size in additional words trained or in hours of audio. BioBERT was trained on additional words of 18 B compared to BERT, and BioClinicalBERT was trained on additional words of 0.5 B compared to BioBERT.

^b^AUC: area under the curve.

^c^Audio recordings were transcribed by Whisper-small. Refer to the Appendix for results from transcription generated by different sizes of the Whisper family models.

^d^BERT: Bidirectional Encoder Representations from Transformers.

### Audio-Based Pipeline

**Figure 3:**
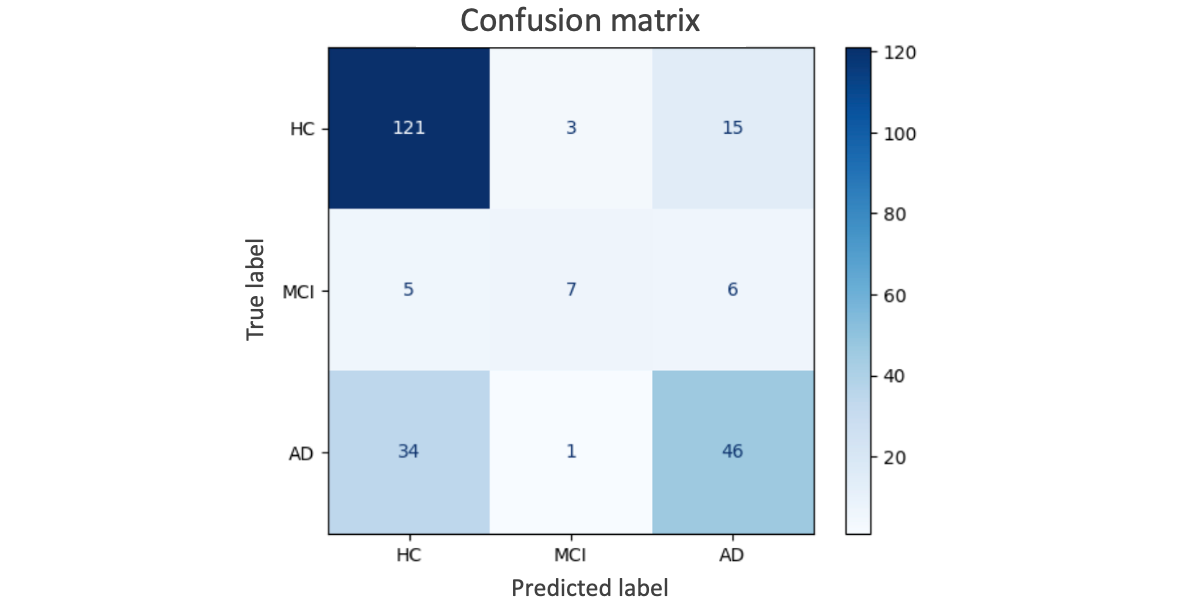
Confusion matrix of the best performing model’s (Whisper-medium) predictions on one replication of the test dataset. AD: Alzheimer disease; HC: healthy controls; MCI: mild cognitive impairment.

Overall, the family of Whisper-based models generally outperformed other audio representation models, likely due to their training on a substantially larger and more diverse multitask dataset (Table 2). Specifically, the medium-sized variant achieved the best performance with an accuracy of 0.731 (SD 0.020) and an AUC of 0.802 (SD 0.014). The 3-class confusion matrix of the best-performing model is presented in Figure 3. Models pretrained on a larger data corpus, such as Whisper, WavLM-base-plus, and WavLM-large, generally outperformed those pretrained on smaller datasets, such as Data2Vec, Wav2Vec, and WavLM-base. Fine-tuning the last layers of Whisper models did not improve performance (Table S4.1 in Multimedia Appendix 1). While performance improved as we scaled the Whisper model from tiny to medium, Whisper-large (1.55 billion parameters, trained on 680,000 hours) performed slightly worse than Whisper-medium (769 million parameters, trained on the same 680,000 hours), suggesting that scaling beyond a certain point does not necessarily lead to better classification performance. One possibility is that the larger model overfits or overspecializes during pretraining and/or fine-tuning, amplifying domain mismatch from pretraining and producing embeddings that are less aligned with dementia-related acoustic or linguistic patterns. Additionally, the higher-dimensional representations generated by Whisper-large may introduce redundancy or noise that dilutes the subtle discriminative cues needed for this clinical task, making them harder for the classification layer to learn the cues from a small, labeled dataset.

Comparing results of both text and audio representation models, text-based models achieved relatively similar performance (0.71-0.73 AUC), likely because they share similar BERT-based architectures, whereas audio-based models exhibited a wider performance range (0.53-0.80 AUC) due to greater diversity in model size and design. Overall, audio-based models outperformed text-based models, and text-based models with pause annotation achieved comparable performance with audio representation models, suggesting that acoustic features provide stronger indicators of AD-related changes than semantic features alone. The strongest-performing audio-based Whisper models likely benefit from their ability to capture both acoustic and semantic information, since their decoder predicts text sequences and therefore their encoder must learn representations from raw audio that implicitly preserve acoustic and semantic context. Whisper’s advantage may also stem from its substantially larger pretraining corpus and greater model capacity, as Whisper-medium is nearly 7 times larger than the text-based models. While the performance of text-based models may also be affected by ASR errors, recent studies have shown that certain types of transcription errors can, in fact, amplify linguistic irregularities associated with cognitive decline, thereby providing additional predictive signal [41,53].

Results from the traditional feature engineering pipeline are presented in Multimedia Appendix 1 (S3). The modern speech and language models significantly outperformed the traditional approach of using hand-engineered acoustic and linguistic features, demonstrating the strength of the recent deep learning models.

### Interpretability Analysis

**Figure 4:**
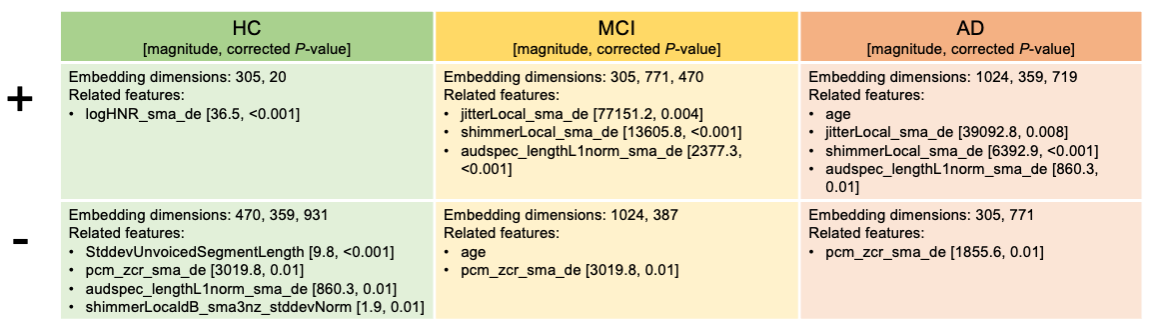
Top contributing embedding dimensions that positively and negatively contribute to health control (HC), mild cognitive impairment (MCI), and Alzheimer disease (AD) classification and their statistically related explainable acoustic/linguistic markers. “+” represents contribution toward the positive direction with respect to the class (increased feature value increases the predicted probability of that class). “–“ represents contribution toward the negative direction with respect to the class (decreased feature value increases the predicted probability of that class). Magnitude equals to the absolute value of the coefficient. *P* value is corrected using false discovery rate correction. Age is directly interpretable from the concatenated feature set.

A visualization of the top contributing embedding dimensions by SHAP is provided in Multimedia Appendix 1 (S7.1). Figure 4 summarizes the acoustic and linguistic markers that have statistically significant relationships with the top 5 embeddings for each class, and the definition of each variable can be found in Multimedia Appendix 2. For HC, the top embeddings are found to have a positive correlation with harmonics-to-noise ratio, which is expected, as a higher harmonics-to-noise ratio reflects clearer, more periodic, and stable vocal production, consistent with preserved laryngeal control and phonatory stability in healthy speakers. Negative correlations with SD of unvoiced segment lengths, zero-crossing rate, L1 norm of auditory spectrum, and SD of shimmer suggest that HC individuals produce more consistent voicing, smoother amplitude variations, and less spectral noise, which fits the literature reporting increased jitter and shimmer in pathological or cognitively impaired speech. For MCI, top contributing embeddings have positive correlations with cycle-to-cycle F0 variation, amplitude variation across cycles, and L1 norm of auditory spectrum, indicating the onset of subtle voice perturbations and increased spectral variability. In AD, embeddings show strong positive correlations with age, jitter, shimmer, and L1 norm of auditory spectrum, alongside negative correlation with zero-crossing rate, confirming the presence of pronounced voice instability, increased aperiodicity, and slower or less articulated speech. Overall, the direction of the correlations aligns with expected physiological and acoustic changes across cognitive decline [21,54]. These results suggest that the high-dimensional embeddings capture rich acoustic and linguistic information that is partially interpretable, extending beyond traditional explainable markers.

## Discussion

### Principal Findings

Our results show that acoustic models generally outperform text-only models in dementia detection, highlighting that nonsemantic speech features are useful indicators for dementia detection, echoed by findings in previous literature [29,30,40,55-57]. Notably, when pauses are explicitly annotated in transcripts, text-based language models achieve performance on par with acoustic models. This finding suggests that among nonsemantic features, pauses are particularly informative, capturing subtle disruptions in speech planning and lexical retrieval that correlate with cognitive impairment.

Pauses have long been recognized in clinical research as a sensitive marker of cognitive decline. Silent-pause frequency and duration differ significantly in individuals with AD compared to HC, suggesting planning and retrieval difficulties [58]. These pausing behaviors correlate with neuropathological biomarkers: for example, longer and more frequent pauses have been linked to tau burden in medial temporal and early neocortical regions [59]. Theoretically, pauses are thought to reflect impairments in cognitive functions that decline early in dementia, such as lexical retrieval, working memory, and speech planning. Reduced cognitive resources or resource depletion (eg, working memory and executive function) may force speakers to insert compensatory pauses during more demanding speech tasks [60]. Pause-related patterns are not only theoretically meaningful but also practically useful: they can be passively captured from spontaneous speech collected in mobile or remote applications, enabling low-cost cognitive screening.

We noticed that using traditional acoustic features alone can achieve prediction accuracy comparable to several foundation models [29,55-57]. However, traditional pipelines rely on manual preselection of acoustic and linguistic features, which may not fully capture the multiple speech characteristics, such as timing, intonation, spectral properties, and pause patterns, that jointly reflect cognitive decline. Some of these discriminative acoustic features manifest as subtle but meaningful deviations from typical speech, which can be difficult to identify or quantify manually, particularly in early-stage disease. In contrast, embedding-based foundation models, particularly those derived from large-scale pretrained speech models, offer greater flexibility and data-driven representation learning. The Whisper-medium model significantly outperformed both the traditional feature-based models and other embedding models, highlighting its strong capacity to capture relevant speech characteristics for ADRD detection without the need for handcrafted features.

Overall, the proposed audio-based AD diagnosis pipeline achieves promising accuracy across the 3 categories. However, the MCI group is significantly underrepresented in our dataset, and models are less accurate and reliable for MCI detection. In reality, the proportion of AD and MCI in the population stands for a different proportion than modeling population in this study. In particular, in the population aged >65 years, we have 29.7% (354/1189) AD and 5.7% (68/1189) MCI in the modeling population. According to the 2025 AD facts and figures, an estimated 7.2 million Americans aged 65 years and older currently live with AD, with approximately 11% aged 65 years and older having AD [61]. There is no accurate MCI estimated proportion in the population, but it is more than doubled than AD across all populations, according to the 2014, 2016, 2018, and 2020 Health and Retirement Study, that all-cause MCI was consistently 23% in each survey [62].

MCI is hard to detect early because the signs are subtle, and there is no simple test for it. Unlike diseases that show clear signals before symptoms, diagnosing MCI and AD relies on recognizing memory and thinking problems, usually first noticed by the person or their family, then confirmed by a doctor using interviews, tests, and sometimes brain scans. Early diagnosis requires noticing small changes in how long it takes people to do everyday tasks or more obvious problems with daily living. Diagnosis is further complicated because so far, there is no single test that can confirm AD or MCI, which makes the process slow and complex. Primary care doctors can do basic memory tests but may feel unprepared to interpret or explain the results, often resulting in referrals and delayed diagnoses when specialists and equipment aren’t locally available [63].

A machine learning–based tool for automatic AD screening via speech holds strong promise for providing an accessible and scalable workflow for early detection. The ability to distinguish HC from individuals with MCI supports early detection, which is critical for timely intervention and monitoring of cognitive decline. Similarly, accurate identification of HC vs AD and MCI vs AD cases enables timely diagnosis and treatment, as the efficacy of current and emerging therapies to slow disease progression is contingent upon early administration (Figure S1.1 in Multimedia Appendix 1) [64]. Participants would be guided through simple spoken tasks, such as describing a picture or narrating a recent experience, with spontaneous speech captured directly through their device’s microphone. The app would process the audio locally or via a secure cloud service using a pretrained foundation speech model (eg, Whisper) to assess cognitive risk. For healthy individuals, periodic screenings, such as once every 3 months, may suffice for proactive monitoring. If a user’s speech is flagged as high risk for MCI, the app would recommend follow-up through an in-clinic cognitive assessment to confirm diagnosis. Early identification of MCI allows for timely intervention, personalized treatment planning, and patient education. Once diagnosed, individuals could continue using the app at a higher frequency (eg, weekly) to monitor their risk score progression and treatment response. This approach supports continuous, personalized cognitive health management in a noninvasive, scalable, and cost-effective manner. By supporting early, low-cost, and remote screening, foundation speech models offer a path toward more accessible Alzheimer caregiving and improved clinical outcomes across diverse real-world environments.

### Comparison With Prior Work

While our best model achieved an accuracy of 0.73, the classification accuracy in AD detection using audio data is slightly higher in several prior studies [28,29,41,42]. Previous study reported an accuracy of 0.68 for eGeMAPS, 0.72 using Wav2Vec2, and 0.73 using data2vec [65], with 1 paper reaching 0.89 [29]. We believe that the difference is due to the diversity of our dataset and the greater complexity of our classification task. Our dataset includes nearly 1200 samples with class imbalance, whereas many prior works on machine learning for AD detection used the Alzheimer’s Dementia Recognition Through Spontaneous Speech 2020 challenge dataset, which is a curated dataset with around 200 samples balanced by age, gender, and diagnosis category [40]. While added variability increases the difficulty of achieving high accuracy, the dataset used in this study reflects a larger and more diverse population, and the type of audio data is also more scalable and easier to obtain in real-world settings. Moreover, most prior studies that achieved higher accuracy focused on a simpler binary classification problem (HC vs AD), while we have a more demanding 3-class classification problem (HC, MCI, and AD). MCI is particularly harder to classify, because it lies on a clinical and biological continuum between normal cognition and AD. The lower accuracy for MCI is also consistent with prior literature [66].

### Limitations

There are several limitations in this study. First, our dataset may not fully represent the broader population in terms of language background, education level, or dialect variation, which could affect acoustic characteristics and thus limit the generalizability of our findings. Second, the MCI class is significantly underrepresented in our dataset. As a result, the model performs more accurately and reliably when distinguishing HC from AD than when detecting MCI. Although we applied a commonly used machine learning strategy, oversampling minority classes in the training data, we observed no meaningful performance improvement. Future studies should aim to collect larger and more balanced samples of MCI participants to enhance both generalizability and clinical use. Additionally, while embeddings extracted from foundation models function as black boxes with limited interpretability. While we conducted post hoc interpretability analysis, more advanced explainability techniques can be explored in the future.

Future research could also explore multilingual models to include non-English samples from the dataset, such as Spanish, Gallego, and Chinese samples. Additionally, incorporating multimodal feature sets, such as participants’ socioeconomics, medical history, and lifestyle information, could provide a more interpretable and comprehensive analysis of the individual’s cognitive status and enhance model performance. As ADRD is a progressive disorder, it would be interesting to collect longitudinal speech data in the future and develop models that are effective at detecting subtle within-subject changes and tracking disease progression over time. Lastly, the study can be extended to examine the stigma associated with ADRD diagnosis, as stigma can significantly deter people from accepting screening or disclosing their status, and the best strategies to enhance the acceptance of audio-based screening tools once they are available.

### Conclusions

Our study presents a comprehensive benchmark for ADRD early detection across several foundation speech and language models and traditional machine learning models on a large, diverse, and clinically relevant dataset. Compared to pretrained language models and more traditional machine learning models, pretrained speech models such as Whisper capture both semantic and nonsemantic acoustic cues that are indicative of cognitive decline and have shown strong predictive performance even without manual transcription. Among the benchmarked speech and language models, Whisper-medium achieved the best performance on this large dataset. Our findings suggest that both semantic and nonsemantic information are crucial for ADRD detection. Future acoustic-based ADRD detection tools should incorporate both semantic and nonsemantic features. The use of acoustic-based automated ADRD detection tools offers a scalable, noninvasive, and cost-effective approach for population-level surveillance that supports earlier intervention and treatment. Technologies such as telehealth and mobile health further enhance their potential for real-world clinical deployment.

## Appendix

Multimedia Appendix 1 Supplementary material: visual overview; clustering of BERT embeddings; additional details for the traditional feature engineering pipeline; additional details for the Whisper family of models; additional details for the interpretability analysis.

Multimedia Appendix 2 Variable definition of 4 groups of acoustic and linguistic features.

## Abbreviations

AD: Alzheimer disease
ADRD: Alzheimer disease and related dementias
ASR: automatic speech recognition
AUC: area under the curve
BERT: Bidirectional Encoder Representations from Transformers
eGeMAPS: Geneva Minimalistic Acoustic Parameter Set
HC: healthy control
IRB: institutional review board
MCI: mild cognitive impairment
PREPARE: Pioneering Research for Early Prediction of Alzheimer’s and Related Dementias EUREKA
SHAP: Shapley additive explanations

## Data Availability

The data used in this study are available through the DementiaBank DrivenData Challenge website [67]. Users may register on TalkBank [37] and DrivenData [68] to request access. The code used for this study is available from the corresponding author upon reasonable request.
