# Supplementary material for: Early Detection of Alzheimer's Disease and Related Dementias From Spontaneous Speech Using Foundation Speech and Language Models: Comparative Evaluation
Source: JMIR Form Res. 2026 May 13;10:e79411. doi: 10.2196/79411 (PMC13216759; doi:10.2196/79411)
Supplement: Multimedia Appendix 1 [file formative_v10i1e79411_app1.docx]

# **Supplementary Material for**

# **Early Detection of Alzheimer’s Disease and Related Dementias from Spontaneous Speech Using Foundation Speech and Language Models: Comparative Evaluation**

## **S 1. Visual overview**


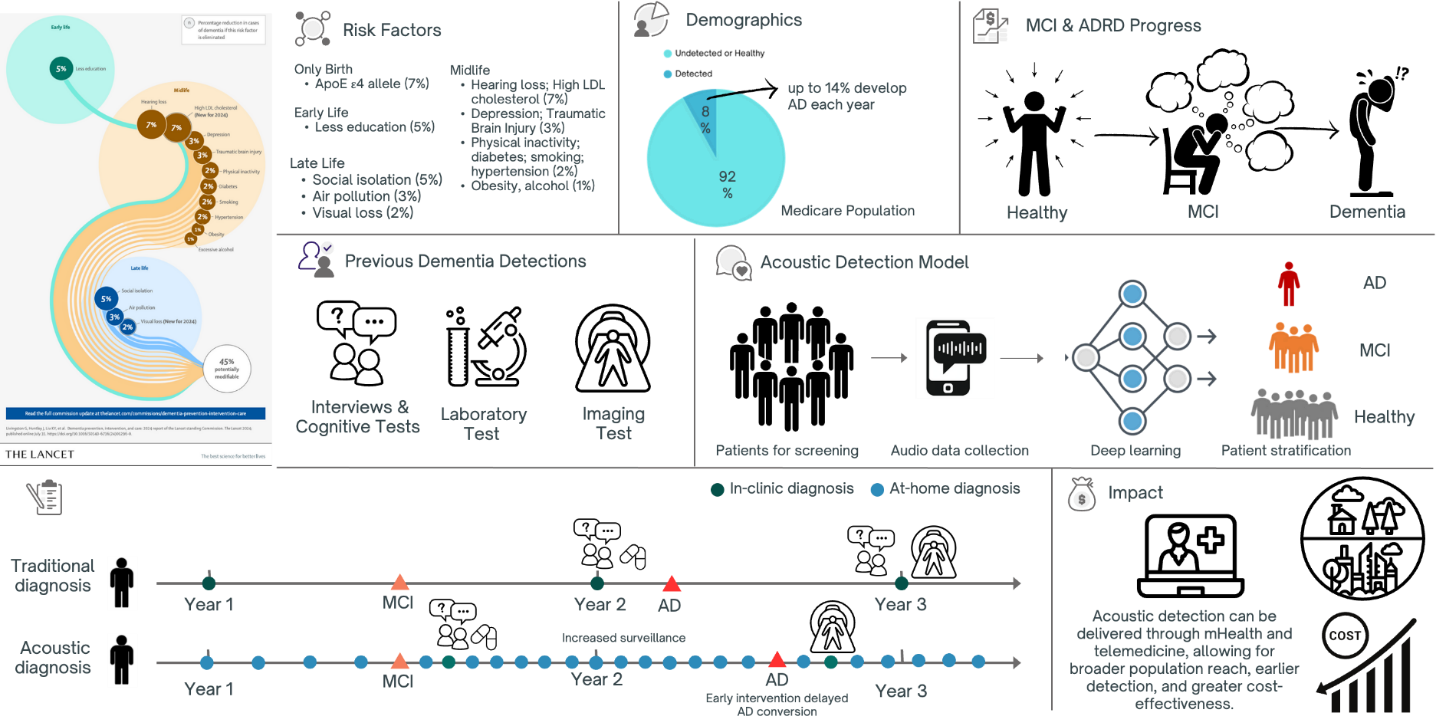


**Figure S1.1**. Progression and detection of Alzheimer's disease and related dementias (ADRD). We illustrate the life-course risk factors for dementia, the progression from healthy aging to mild cognitive impairment (MCI) and Alzheimer’s disease (AD), and the potential of acoustic detection as an innovative diagnostic tool. In the acoustic-based diagnosis framework, individuals provide spontaneous speech recordings via a mobile device or with clinician assistance. Pre-trained deep learning models extract acoustic embeddings from speech, which are then used to classify the individual’s cognitive status as healthy, MCI, or AD. The model outputs can recommend an in-clinic follow-up for formal diagnosis and potential intervention. This framework enables convenient, flexible, and scalable surveillance for early detection of ADRD.

**S 2.** **Clustering of BERT embeddings for topic modelling**


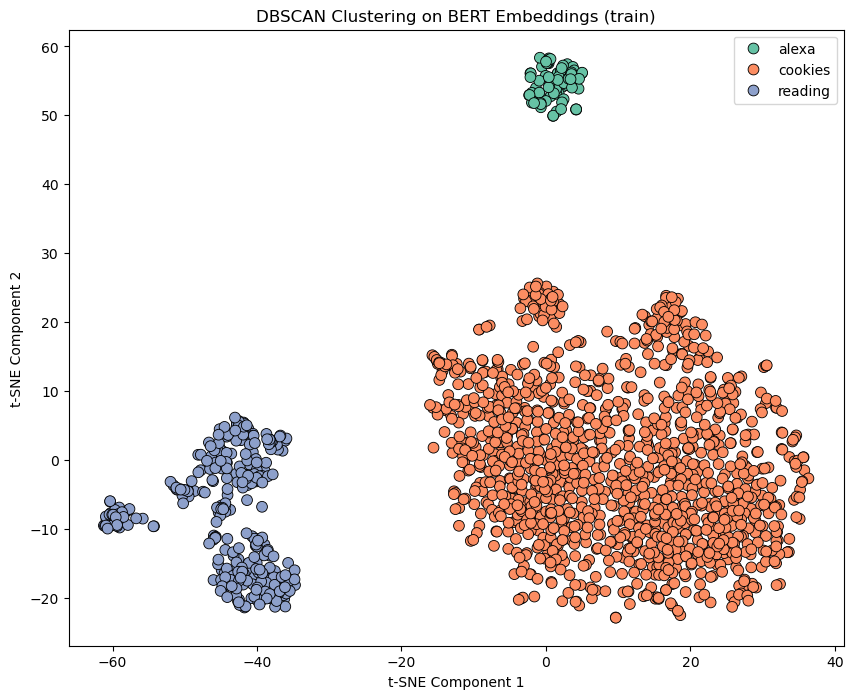


**Figure S2.1.** t-SNE visualization of clustering results on BERT embeddings of the original PREPARE Challenge dataset. Human evaluation classified the clusters into those performing spontaneous speech task (Cookies Theft Picture) and other sentence reading tasks.

**S 3. Traditional Feature Engineering Pipeline**

## **S 3.1. Methods**

**Audio preprocessing:** Each raw audio clip (48 kHz) is resampled to 16 kHz since high frequencies in the audio recordings typically do not contain speech. Audio amplitudes were normalized and beginning and end silence were trimmed. DeepFilterNet was used to remove background noise.

**Feature extraction:** We extracted multiple feature sets to capture acoustic and linguistic characteristics of speech. Acoustic features were computed using the openSMILE toolkit, including the extended Geneva Minimalistic Acoustic Parameter Set (eGeMAPS) [1], which captures prosodic, spectral, and voice quality features relevant to affective and clinical speech analysis, and the ComParE feature set [2], which provides a large-scale brute-force representation of low-level descriptors and functionals. In addition, 20-dimensional Mel-Frequency Cepstral Coefficients (MFCCs) [3] were computed to characterize the spectral envelope of the speech signal. For linguistic analysis, we used Linguistic Inquiry and Word Count (LIWC) to extract psychologically meaningful lexical features [4]. All features were standardized prior to modeling.

**Classification Models:** Each feature set was used independently to train binary classification models distinguishing Alzheimer’s disease (AD) patients from cognitively normal controls. We employed PyCaret [5] to compare the model performance across multiple machine learning models including Extreme Gradient Boosting (XGBoost), Gradient Boosting Machine (GBM), Light GBM, Random Forest, Ada Boost Classifier, Ridge Classifier, Logistic Regression, Extra Trees, Decision Tree, K Neighbors Classifier, Linear Discriminant Analysis, Naive Bayes, and SVM. Hyperparameters were tuned via grid search using 5-fold cross-validation. The best performing model was chosen based on cross-validation AUC and the best model’s test performance across five independent replications was reported.

## **S 3.2. Results**

### Traditional Pipeline

**Table S3.1** summarizes the results of traditional feature engineering pipelines across five repetitions. Among all traditional features, the eGeMAPS achieved the highest accuracy of 0.643 (±0.020) and an AUC of 0.767 (±0.013) – performance comparable to several foundation speech models such as Unispeech, WavLM, Wav2Vec2, and Data2Vec.

**Table S3.1**. Test results for traditional feature engineering pipeline across 5 replications

| Feature | Model | Accuracy | AUC^a^ |
| --- | --- | --- | --- |
| eGeMaps^b^ | Extra Trees | *0.643 (0.020)* | *0.767 (0.013)* |
| ComParE | Random Forest | 0.588 (0.021) | 0.677 (0.012) |
| MFCC^c^ | Random Forest | 0.635 (0.020) | 0.754 (0.013) |
| LIWC^d^ | Logistic Regression | 0.583 (0.013) | 0.639 (0.023) |

^a^AUC: area under the curve.

^b^eGeMaps: Geneva Minimalistic Acoustic Parameter Set

^c^MFCC: Mel-Frequency Cepstral Coefficients

^d^LIWC: Linguistic Inquiry and Word Count

**S 4.** **Results of Whisper models with fine-tuning**

**Table S4.1.** Test results for Whisper family of models across 5 replications with fine tuning

| **Models** | **Accuracy** | **AUC**^a^ |
| --- | --- | --- |
| Whisper-tiny.en | 0.680 (0.034) | 0.761 (0.017) |
| Whisper-base.en | 0.709 (0.026) | 0.789 (0.010) |
| Whisper-small.en | 0.715 (0.036) | 0.800 (0.025) |
| Whisper-medium.en | **0.723** (0.035) | **0.802** (0.028) |
| Whisper-large | 0.698 (0.019) | 0.784 (0.017) |
| ^a^AUC: area under the curve.  Last layers of Whisper models were fine-tuned while other layers were kept frozen during training. | | |

**S 5.** **Whisper models with balanced sampling**

Due to imbalanced class distribution in our modeling dataset, we conducted a balanced oversampling procedure on our training data on our best performing model (Whisper-medium). Specifically, we assigned each sample a weight inversely proportional to the number of observations in its class and then resampled the training data according to these weights. This procedure produces a dataset with balanced class while maintaining the original training set size by oversampling the minority classes (MCI and AD in our case). We then retrained our model using this oversampled dataset. The average accuracy (standard deviation) and AUC (standard deviation) after oversamling are 0.691 (0.021) and 0.809 (0.005), respectively.

**S 6.** **Whisper-Small and Whisper-Medium for transcription**


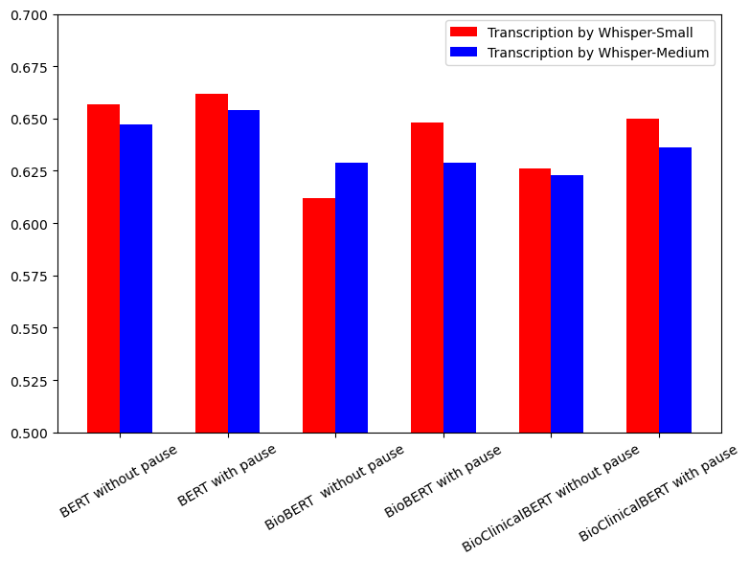

**Figure S6.1.** Average accuracy of text-based models when Whisper-small and Whisper-medium are used for transcription.

**S 7.** Interpretability Analysis


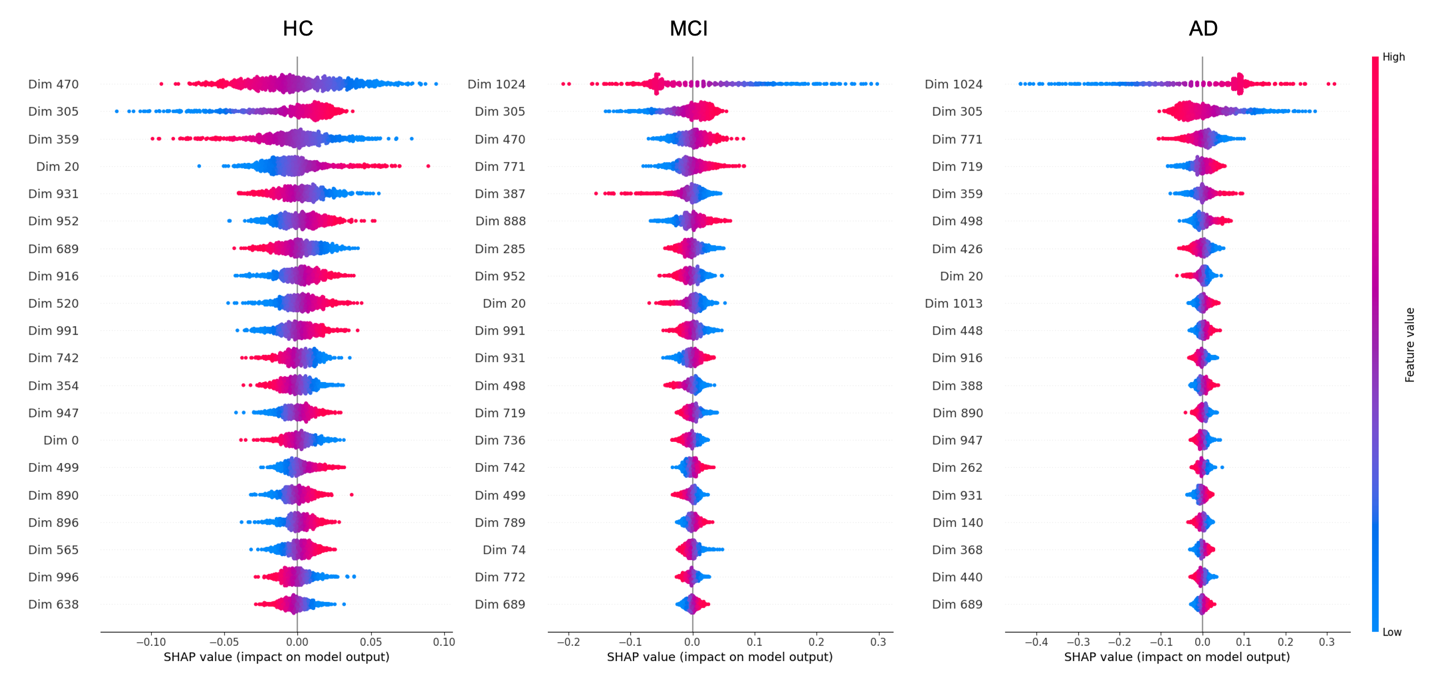


**Figure S7.1.** Top contributing embedding dimensions for HC, MCI, and AD classes based on Whisper-medium by SHAP. Dimension 1024 corresponds to Age. Abbreviations: SHapley Additive exPlanations (SHAP), Healthy Controls (HC), Mild Cognitive Impairment (MCI), Alzheimer’s Disease (AD).

**References**

1. F. Eyben, K.R. Scherer, B.W. Schuller, J. Sundberg, et al. The Geneva minimalistic acoustic parameter set (GeMAPS) for voice research and affective computing. IEEE transactions on affective computing. 2015. **7**(2): pp. 190-202. [https:// ieeexplore.ieee.org/document/7160715](https://ieeexplore.ieee.org/document/7160715).

2. B. Schuller, S. Steidl, A. Batliner, A. Vinciarelli, et al. The INTERSPEECH 2013 computational paralinguistics challenge: Social signals, conflict, emotion, autism. 2013. <https://eprints.gla.ac.uk/93665/1/93665.pdf>.

3. B. McFee, C. Raffel, D. Liang, D.P. Ellis, et al. librosa: Audio and music signal analysis in python. SciPy. 2015. **2015**: pp. 18-24. [https://www.academia.edu/ download/40296500/librosa.pdf](https://www.academia.edu/download/40296500/librosa.pdf).

4. J.W. Pennebaker, M.E. Francis. R.J. Booth. Linguistic inquiry and word count: LIWC 2001. Mahway: Lawrence Erlbaum Associates. 2001. **71**(2001): pp. 2001. <http://downloads.liwc.net.s3.amazonaws.com/LIWC2015_OperatorManual.pdf>.

5. M. Ali, PyCaret: An open source, low-code machine learning library in Python. 2020.
